# Food dye staining of dental composites: material variability and mouthwash-induced staining reversibility

**DOI:** 10.2340/biid.v13.45823

**Published:** 2026-04-24

**Authors:** Burçin Deniz, Osman Tolga Harorlı

**Affiliations:** Department of Restorative Dentistry, Faculty of Dentistry, Akdeniz University, Antalya, Turkey

**Keywords:** resin based composites, food colouring agents, colour stability, mouthwashes, spectrophotometry

## Abstract

**Objective:**

Food dyes, widely used in consumables, may interact with polymeric materials, yet their staining effects on resin based dental composites remain underexplored. This *in vitro* study investigates the staining potential of five common water-soluble food dyes on contemporary dental composites and evaluates the efficacy of Listerine Advanced White mouthwash in reversing discolouration, using both colour difference and whiteness-based analyses.

**Methods:**

Five synthetic dyes – Tartrazine, Sunset Yellow, Ponceau 4R, Carmoisine, and Brilliant Blue – were tested on six resin composites: Charisma Smart, Estelite ∑ Quick, Filtek One Bulkfill, RubyFlow, Spectra SphereTEC High Viscosity (STHV), and Filtek Ultimate. Sixty disk-shaped samples (7 mm × 2 mm) per composite (*N* = 360) were immersed in dye solutions (50 mg/L) or distilled water (control) for 7 days. Colour changes were evaluated using the CIEDE2000 colour difference formula (ΔE₀₀) and the dental whiteness index (WI_D_), calculated from Commission Internationale de l’Eclairage L*a*b* color space (CIELAB) coordinates obtained with a spectrophotometer.

**Results:**

Brilliant Blue exhibited the highest staining potential, producing ΔE₀₀ values exceeding the clinically unacceptable threshold (1.8) in all composites except Spectra STHV, and was also associated with the greatest changes in WI_D_ values. Other dyes caused variable and generally less pronounced changes in both ΔE₀₀ and WI_D_. The whitening mouthwash partially reversed discolouration, reflected by significant increases in WI_D_ and reductions in ΔE₀₀, with significant recovery observed in Estelite, RubyFlow, and Filtek One (*p* < 0.05). Analysis of variance revealed significant effects of dye type, composite material, and treatment on both ΔE₀₀ and WI_D_ outcomes (*p* < 0.001).

**Conclusion:**

The colour stability and whiteness of dental resin composites are affected by synthetic food dyes in a material- and dye-dependent manner. Whitening mouthwash can partially restore both colour difference and whiteness; however, complete recovery to baseline values is generally not achieved.

## Introduction

Dental resin composites, widely employed in modern dentistry for their aesthetic versatility and durability, have become a cornerstone of restorative and cosmetic procedures [1]. The susceptibility of dental composite restorations to staining is a significant clinical concern [2, 3]. With the increasing demand for natural-looking restorations, understanding and mitigating discolouration is essential to ensure long-term aesthetic success and patient satisfaction [4–6].

Food dyes are ubiquitous in modern diets, widely used to enhance the visual appeal of food and beverages [5, 6]. However, their pervasive presence raises concerns for dental composites, as these artificial colourants may cause staining or discolouration, compromising the aesthetic outcomes of restorations.

Currently, there are limited data on the staining potential of common food dyes on dental composites. This knowledge gap hinders our understanding of how specific food dyes interact with different composite materials, potentially leading to unexpected discolouration of restorations [6, 7]. It remains unclear whether these products can effectively reverse staining caused by food dyes on composite restorations.

Whitening mouthwashes have been increasingly used as a non-invasive approach for managing extrinsic discolouration due to their affordability and ease of use [7, 8]. Unlike professional bleaching agents that rely on oxidative mechanisms, many over-the-counter whitening mouthwashes are hydrogen peroxide–free and act primarily through chemical stain control [9]. Formulations containing polyphosphates, such as pyrophosphates and sodium hexametaphosphate, function by binding to calcium ions on the tooth or restorative surface, thereby inhibiting chromogen adhesion and facilitating the removal of superficial stains [9, 10] These agents primarily target surface discolouration rather than inducing intrinsic whitening, and their effectiveness may therefore depend on the nature of the stain and the characteristics of restorative materials [9].

This study aims to address this knowledge gap by evaluating the effectiveness of a whitening mouthwash in reversing food dye-induced staining on common dental composite materials.

To address these questions, we propose the following hypotheses:

- H01 There is no significant difference in the staining potential of different food dyes on dental resin composites.
- H02 There is no difference in the colour stability of tested dental resin composites when stained with various food dyes.
- H03 Treatment with whitening mouthwashes does not significantly affect the colour stability of dental resin composites stained with food dyes, compared to untreated stained controls.
- H04 Whitening mouthwash treatment does not significantly alter the Whiteness Index for Dentistry (WI_D_) of dental resin composites stained with food dyes.

## Materials and methods

In this *in vitro* study, six commercially available A2-shade resin composites were selected with varying properties, encompassing a range of particle sizes, inorganic filler contents, and resin matrix structures. Specific characteristics of each composite are detailed in Table 1.

**Table 1:** Composition and characteristics of resin-based composite materials.

Material name	Material type	Filler particle size (range and type)	Filler content (weight % / volume %)	Chemical composition	Batch / Lot number	Unique features / Technologies	Manufacturer information
**Charisma Smart (CHR)**	Submicron-hybrid	0.005–10 μm Barium Aluminium Boro Fluor Silicate Glass	78 wt.% / 59 vol.%	Bis-GMA, TEGDMA, UDMA, Camphorquinone	M010538	Microglass Color-Stabilizing Technology	Kulzer GmbH, Hanau, Germany
**Estelite ∑ Quick** **(EST)**	Sub-micron	0.2 μm Monodispersing Spherical Silica-Zirconia Filler	82 wt.% / 71 vol.%	Bis-GMA, TEGDMA, UDMA, Camphorquinone	E8662	RAP Photopolymerization Initiator Technology	Tokuyama Dental, Tokyo, Japan
**One Bulk Fill Restorative (OBF)**	Bulk Fill	20 nm Silica, 4–11 nm Zirconia, 4–20 Nm Zirconia/Silica Clusters, 100 nm Ytterbium Trifluoride (Ybf3)	76.5 wt.% / 58.5 vol.%	AUDMA, AFM, Diurethane-DMA, DDDMA (1,12-Dodecanediol dimethacrylate)	NE55682	AFM (Addition-Fragmentation Monomer), AUDMA (Aromatic Urethane Dimethacrylate)	3M ESPE, MN, USA
**RubyFlow (RBF)**	Micro-hybrid	0.02–0.7 μm Barium Glass, Nanosilica Fillers	62 wt.%	Bis-GMA, TEGDMA	RFA270	Flowable	Ruby Dent, İstanbul, Turkey
**Spectra STHV (SPC)**	Nano-ceramic	Spherical 15 μm PPRD particles, Barium Aluminum Borosilicate Glass, Ytterbium Fluoride	78–80 wt.% / 60–62 vol.%	Polysiloxane Methacrylate Dimethacrylate, Bis(4-methylphenyl) Iodonium Hexafluorophosphate, Ethyl-4 (dimethyl amine) Benzoate, UV Stabilizer, Camphorquinone	0643	SphereTEC^®^ Spherical Filler Technology, PPRD (Submicron Pre-Polymerized Resin Filler Glass Particles)	Dentsply Sirona, Konstanz, Germany
**Ultimate Universal Restorative (ULT)**	Nanofill	20 nm Silica, 4–11 nm Zirconia, 0.6–10 Μm Zirconia/Silica Clusters	78.5 wt.% / 63.3 vol.%	Bis-GMA, UDMA, TEGDMA, PEGDMA, Bis-EMA	NE14463	3M’s TRUE Nanotechnology	3M ESPE, MN, USA

For each resin composites, 60 disc-shaped samples (*n* = 60) were prepared, totalling 360 samples (*N* = 360). Each sample, measuring 7 mm in diameter and 2 mm in thickness, was fabricated using a Teflon mould. The resin composite was carefully inserted into the mould to eliminate air gaps, and excess material was removed using a transparent Mylar strip (Type D, DuPont, DE, USA).

Samples were cured using an Light-Emitting Diode (LED) curing device (Valo, Ultradent, South Jordan, UT, USA) at 1000 mW/cm² for 20 seconds per surface, following the manufacturer’s recommendations. The LED curing device’s radiation output was verified before each curing session using a radiometer (SDS Kerr, Orange, CA, USA). No additional finishing or polishing procedures were performed. This approach was intentionally selected to standardise surface topography and eliminate variability associated with polishing systems, which are known to influence surface roughness and, consequently, stain susceptibility. After preparation, all samples were immersed in distilled water (pH 7.27) at room temperature for 24 hours.

The study utilised five commonly used water-soluble synthetic food dyes, with specific characteristics detailed in Table 2. To ensure consistency, dye solutions were prepared at the maximum allowable concentration for non-alcoholic beverages (50 mg/L) [11]. Precisely 25 mg of each powdered dye was weighed using a precision scale (Shimadzu AP225WD Semi-Micro Balance, Shimadzu Corporation, Kyoto, Japan) and dissolved in 500 mL of deionised water.

**Table 2:** Food additives and their properties.

Common name	E code (European Union)	FD&C code (USA)	Colour index number	Colour description	Chemical classification	Applications	Acceptable daily intake (mg/kg)
**Brilliant Blue (BR)**	E133	Blue No:1	42090	Blue	Triphenylmethane	Food, cosmetics, pharmaceuticals, dietary supplements	10 mg/kg
**Carmoisine (CA)**	E122	Red No. 10	14720	Dark Red	Monoazo	Specialty foods (cheese, dried fruits), alcoholic beverages, pharmaceuticals	4 mg/kg
**Ponceau 4R (PO)**	E124	Not Applicable	16255	Strawberry Red	Monoazo	Food	0.7 mg/kg
**Sunset Yellow (SU)**	E110	Yellow 6	15985	Orange	Monoazo	Food, cosmetics, pharmaceuticals	2.5 mg/kg
**Tartrazin (TA)**	E102	Yellow 5	19140	Lemon Yellow	Monoazo	Food, cosmetics, pharmaceuticals, textiles	7.5 mg/kg

The 360 composite samples were evenly distributed into 36 groups (*n* = 10 per group), representing each combination of composite resin and dye solution, including a control group for each resin. After a 24-hour immersion in distilled water, the samples were dried with absorbent papers, and their initial colour values (T0) were measured using a Vita Easyshade spectrophotometer (VITA Zahnfabrik GmbH, Bad Sackingen, Germany) against a standard black background.

Following the initial colour measurements (T0), the control groups remained immersed in distilled water (pH 7.27), while the remaining groups were submerged in their respective dye solutions (CN: Control, Br: Brilliant Blue Ca: Carmoisine, Po: Ponceau, 4R Su: Sunset Yellow, Ta: Tartrazine) for 1 week (7 days), with fresh dye solutions provided daily. After 7 days, all samples were rinsed thoroughly under running water for 60 seconds. Once dried, their colour changes were quantified using a spectrophotometer based on L*, a*, and b* coordinates, resulting in ΔE₀₀ values (T1).

After the staining period (T1), all groups were immersed in Listerine Advanced White mouthwash for 1 week (7 days), replacing the solution daily. The mouthwash’s composition included aqua, sorbitol, tetrapotassium pyrophosphate, pentasodium triphosphate, citric acid, Poloxamer 407, and active/flavouring agents such as eucalyptol, thymol, menthol, and sodium fluoride (220 ppm F^–^). After 1 week, the samples were rinsed under running water for 60 seconds and dried. The average ΔE values for each group were calculated (T2) to assess the mouthwash’s effect on removal of staining.

Colour shifts were assessed using the CIEDE2000 formula, with perceptible colour changes defined as ΔE₀₀≥ 0.8 and clinically acceptable changes as ΔE₀₀ ≤ 1.8 [12].


$$E_{00}=\sqrt{{\frac{∆L\prime }{K_{L}S_{L}}}^{2}+{\frac{∆C\prime }{K_{C}S_{C}}}^{2}+{\frac{∆H\prime }{K_{H}S_{H}}}^{2}+R_{T}\;\;\frac{∆C\prime }{K_{C}S_{C}}\frac{∆H\prime }{K_{H}S_{H}}}$$

The colour variations were illustrated by transforming the lab values into RGB values using a Python script. This script leveraged the matplotlib library to generate colour swatches that visually represented the recorded lab values. The conversion process included the following steps:

The lab values were transformed into XYZ coordinates using standard conversion formulas, incorporating adjustments to account for non-linear values.

Y = (L + 16) / 116

X = a × 0.002 + Y

Z = Y – b × 0.005

The XYZ values were converted into RGB values by applying the following transformation matrix:

[R] [3.2406 –1.5372 –0.4986] [X]

[G] = [–0.9689 1.8758 0.0415] [Y]

[B] [0.0557 –0.2040 1.0570] [Z]

The RGB values were modified through gamma correction to ensure accurate colour representation.

R = 1.055 × R^(1/2.4) – 0.055 if R > 0.0031308 else 12.92 × R

G = 1.055 × G^(1/2.4) – 0.055 if G > 0.0031308 else 12.92 × G

B = 1.055 × B^(1/2.4) – 0.055 if B > 0.0031308 else 12.92 × B

Finally, the RGB values were constrained within the range [0, 1] to maintain valid colour representation.

The whiteness of the resin composite specimens was quantified using the dental whiteness index (WI_D_), which is a customised index developed specifically for dentistry and based on the CIELAB colour space. Colour measurements were obtained using a spectrophotometer, and the corresponding Commission Internationale de l’Eclairage (CIE) L*, a*, and b* values were recorded for each specimen at baseline (T0), after staining (T1), and after whitening (T2).

The WI_D_ values were calculated according to the formula proposed by Pérez et al. [13].


WI_D=0.511L*−2.324a*−1.100b*

where L* represents lightness, a* the red–green axis, and b* the yellow–blue axis.

Higher WI_D_ values indicate increased perceived whiteness, whereas lower values reflect darker or more chromatic appearances.

Data were analysed using Jamovi software (version 2.3.28.0, The Jamovi project, Australia). Normality was assessed using skewness and kurtosis tests. Repeated-measures analysis of variance (ANOVA) was used to evaluate the effects of various factors on colour change, followed by Tukey’s post-hoc test for pairwise comparisons. A significance level of α = 0.05 was used for all analyses.

## Results

Statistical analysis using repeated measures ANOVA revealed significant differences in colour changes across the T0–T1–T2 time points (*p* < 0.001). In addition, significant effects of composite brands (*p* = 0.004) and food dyes (*p* < 0.001) on colour changes were observed. However, the interaction between time points (T0–T1–T2), composite brands, and food dyes did not demonstrate a statistically significant effect on colour changes (*p* = 0.186).

**Figure 1:**
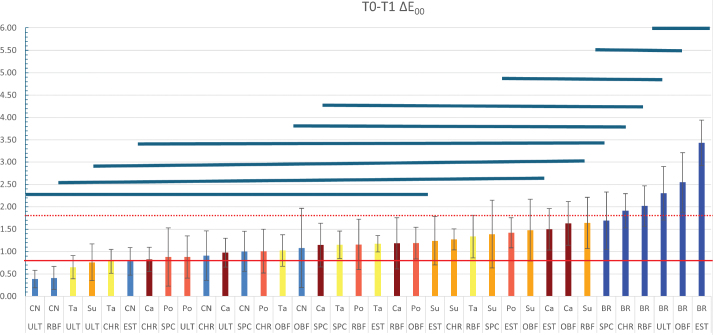
The colour change of resin composites following immersion in dye solutions. The solid red line represents the visually perceptible threshold (ΔE_00_ = 0.8), while the dashed red line indicates the clinically acceptable threshold for colour change (ΔE_00_ = 1.8). Bar graphs below the blue lines represent groups that showed no statistically significant differences in colour change. CN: Control; Br: Brilliant Blue; Ca: Carmoisine; Po: Ponceau; 4R Su: Sunset Yellow; Ta: Tartrazine; CHR: Charisma Smart; EST: Estelite ∑ Quick; OBF: One Bulk Fill Restorative; RBF: RubyFlow; SPC: Spectra STHV; ULT: Ultimate Universal Restorative.

At baseline (T0), all composite samples displayed high L* values (ranging approximately from 70 to 85), indicating a light appearance (Figure 1). Composite brands Spectra STHV (SPC), Charisma Smart (CHR), and Ultimate Universal Restorative (ULT) showed higher initial L* values compared to other composites. The a* values were generally stable and close to zero or within a narrow range, reflecting neutral or slightly red/green-tinted initial colours. In contrast, b* values were typically above zero, suggesting a slight yellow tint, with variations among composite brands.

Exposure to food dyes at T1 resulted in notable colour shifts across all composite samples, varying by dye type and composite brand. Br caused a marked decrease in b* values (a shift toward blue) and a reduction in L* (indicating darkening). Red and yellow dyes (Ca, Po, Su, and Ta) induced variable changes in a* (toward red or green) and b* (toward yellow or blue), with generally minor shifts in L* values across all brands, resulting in subtle colour alterations due to staining.

**Figure 2:**
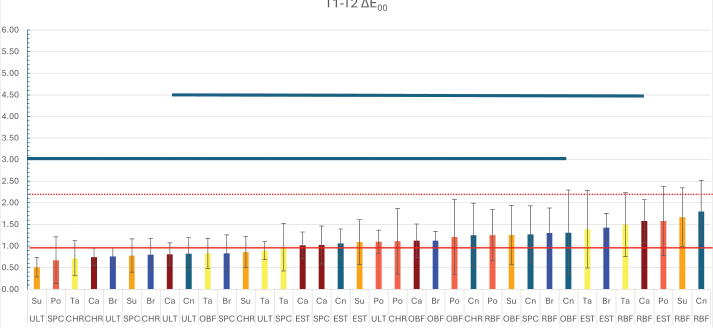
The colour change of stained resin composites following immersion in whitening mouthwash. The solid red line represents the visually perceptible threshold (ΔE_00_ = 0.8), while the dashed red line indicates the clinically acceptable threshold for colour change (ΔE_00_ = 1.8). Bar graphs below the blue lines represent groups that showed no statistically significant differences in colour change. CN: Control; Br: Brilliant Blue; Ca: Carmoisine; Po: Ponceau; 4R Su: Sunset Yellow; Ta: Tartrazine; CHR: Charisma Smart; EST: Estelite ∑ Quick; OBF: One Bulk Fill Restorative; RBF: RubyFlow; SPC: Spectra STHV; ULT: Ultimate Universal Restorative.

The colour change (ΔE₀₀) of resin composites following immersion in dye solutions was assessed from baseline (T0) to after staining (T1). The ΔE₀₀ values varied significantly across composite brands and dye types, as shown in Figure 2. The visually perceptible threshold (ΔE₀₀ = 0.8, solid red line) and the clinically acceptable threshold (ΔE₀₀ = 1.8, dashed red line) were used to evaluate colour stability. Following immersion in dye solutions, maximum discolourations in composite resins were observed in groups exposed to Br. All composite groups immersed in Br exhibited colour changes above the clinically acceptable threshold (ΔE₀₀ = 1.8), except for the SPC composite group. The ULT, One Bulk Fill Restorative (OBF), and Estelite ∑ Quick (EST) composites in Br solution demonstrated statistically significant discolourations (*p* < 0.05). In contrast, all composite groups immersed in distilled water (control) displayed colour changes below the acceptable threshold (ΔE₀₀ = 1.8). However, the OBF, SPC, and CHR groups in the in distilled water showed changes exceeding the visually perceptible threshold (ΔE₀₀ = 0.8).

Whitening with mouthwash at T2 partially reversed the discolouration observed at T1, with L*, a*, and b* values trending toward baseline levels (T0). However, a complete recovery was not achieved for all samples. Br-stained samples generally retained residual negative shifts in b* (blue tint), indicating incomplete removal of blue pigmentation (Figure 1). Samples exposed to red and yellow dyes (Ca, Po, Su, Ta) showed better recovery in a* and b* values, though minor residual shifts persisted, particularly in OBF and RubyFlow (RBF). SPC and ULT demonstrated superior colour recovery, with L*, a*, and b* values closer to baseline across all dyes, suggesting greater resistance to permanent staining or enhanced responsiveness to whitening.

**Figure 3:**
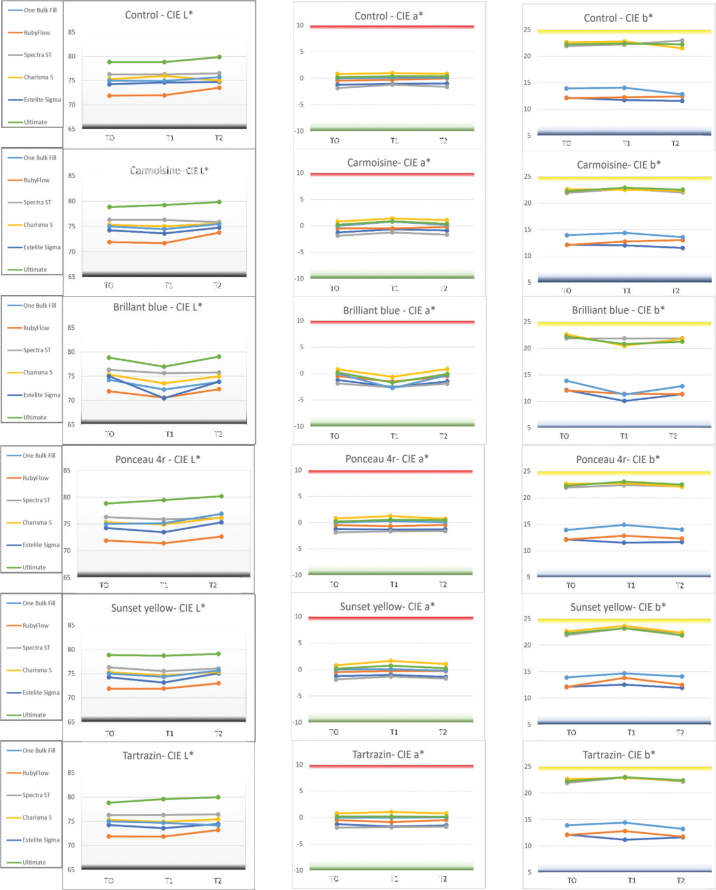
Changes in CIE colour coordinates over time at three measurement points: baseline (T0), after dye immersion (T1), and following treatment with whitening mouthwash (T2). Each chart represents the evolution of L*, a*, and b* values for different resin composites. CN: Control; Br: Brilliant Blue; Ca: Carmoisine; Po: Ponceau 4R; Su: Sunset Yellow; Ta: Tartrazine. Resin composites: CHR: Charisma Smart; EST: Estelite ∑ Quick; OBF: One Bulk Fill Restorative; RBF: RubyFlow; SPC: Spectra STHV; ULT: Ultimate Universal Restorative.

Colour change values (ΔE₀₀) between T1 and T2 are presented in Figure 3. Lower ΔE₀₀ values indicate a diminished colour recovery effect of the whitening mouthrinse, whereas higher values correspond to more pronounced colour changes. Statistical analysis revealed that the whitening mouthrinse had a significantly greater effect on the RBF-CN, RBF-SU, and EST-PO groups (*p* < 0.05). At T2, whitening partially mitigated the discolouration, showing significant recovery effects in EST, RBF, and OBF composites (*p* < 0.05).

**Figure 4:**
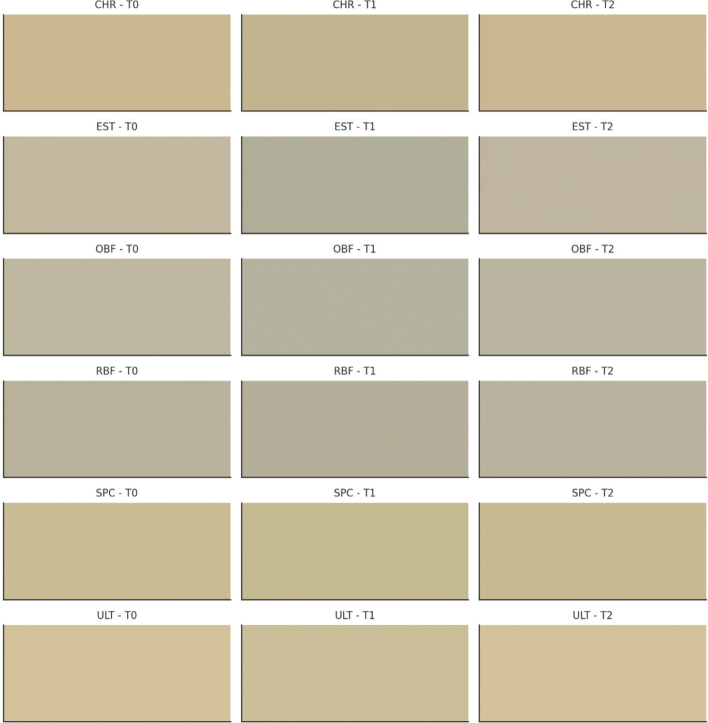
Visual representation of colour changes in resin composites in Br (Brilliant Blue) over time. Each row corresponds to a different resin composite, and columns represent three time points: baseline (T0), after dye immersion (T1), and after treatment with whitening mouthwash (T2). The resin composites included are Charisma Smart (CHR), Estelite ∑ Quick (EST), One Bulk Fill Restorative (OBF), RubyFlow (RBF), Spectra STHV (SPC), and Ultimate Universal Restorative (ULT). This visualization highlights the impact of staining and subsequent whitening treatment on resin composite colour stability.

Figure 4 illustrates the effect of Br, which led to significant colour changes in various composite resins across three time points: T0, T1, and T2. Each row represents a different composite resin, while the columns display the progression of colour changes over time. This visual depiction emphasises the pronounced impact of dyes, making the colour variations clearly noticeable.

**Figure 5:**
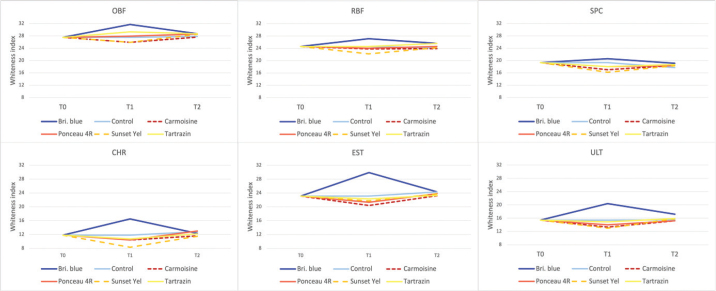
Changes in the dental whiteness index (WI_D_) of the resin composites OBF, RBF, SPC, CHR, EST, and ULT across the three evaluation periods: baseline (T0), after staining (T1), and after whitening (T2). Each panel represents a single composite, and coloured lines indicate the different staining solutions (Brilliant Blue, Ponceau 4R, Carmoisine, Sunset Yellow, Tartrazin, and Control). Higher WI_D_ values indicate greater perceived whiteness, whereas lower values reflect increased discolouration. CHR: Charisma Smart; EST: Estelite ∑ Quick; OBF: One Bulk Fill Restorative; RBF: RubyFlow; SPC: Spectra STHV; ULT: Ultimate Universal Restorative.

The repeated-measures ANOVA demonstrated that WI_D_ values were significantly influenced by experimental stage, staining solution, and resin composite, with significant interactions among these factors (*p* < 0.001) (Figure 5).

At baseline (T0), WI_D_ values differed significantly among the tested composites, reflecting differences in initial whiteness. Following the staining procedure (T1), a general decrease in WI_D_ was observed for most staining solutions, indicating increased discolouration. This reduction was more pronounced in certain composites, particularly CHR and ULT, whereas OBF and EST demonstrated relatively higher WI_D_ values and greater resistance to whiteness loss (Figure 5).

Among the staining solutions, Carmoisine and Sunset Yellow caused the greatest reductions in WI_D_ across most composites, while Brilliant Blue resulted in comparatively higher WI_D_ values at T1 in several materials.

After the whitening procedure (T2), WI_D_ values increased significantly in all experimental groups compared with T1 (*p* < 0.05), indicating partial recovery of whiteness. However, in most composite–staining combinations, WI_D_ values at T2 did not fully return to baseline levels. The extent of whitening recovery varied depending on the composite material, with OBF and EST showing greater restoration of WI_D_, whereas CHR and ULT exhibited lower final whiteness values.

## Discussion

This study evaluated the staining behaviour of five water-soluble synthetic food dyes on six contemporary resin composites and assessed whether a polyphosphate-containing whitening mouthwash could reverse dye-induced discolouration. The main findings were that (1) staining was strongly dye-dependent, with Brilliant Blue producing the largest colour differences and the greatest losses in whiteness in most materials; (2) staining magnitude varied substantially among composites, indicating material-dependent susceptibility; and (3) 1 week of whitening mouthwash exposure produced statistically significant, but incomplete, recovery in both ΔE00 and WI_D_.

Across materials, Brilliant Blue was the biggest chromogenic challenge in terms of ΔE00 and WI_D_ loss, and in most composites staining exceeded the clinically acceptable threshold (ΔE₀₀ = 1.8) (Figure 1). This aligns with previous reports that Brilliant Blue-based plaque-staining agents can produce visible discolouration in other dental materials/appliances, supporting the notion that Brilliant Blue can behave as a highly relevant chromogen in the oral environment [14]. The associated negative shift in b* (toward blue) and reduction in L* (Figure 3) suggests that blue pigmentation was not only adsorbed on the surface but also contributed to an overall darkening effect, which is clinically meaningful because perceived tooth colour is strongly driven by both lightness and chroma components [15]. In contrast, yellow and red dyes generally produced smaller and more variable changes, consistent with weaker affinity for the composite surface and/or reduced penetration into the resin matrix.

Several mechanisms may explain the pronounced effect of Brilliant Blue in resin composites. Dyes differ in molecular size, polarity, and ionic character, which can influence diffusion into water plasticised resin networks and adsorption to filler–matrix interfaces. In addition, dye–polymer interactions (e.g. hydrogen bonding with polar monomers and additives) may favour retention of specific chromogens. Although the present design does not isolate these mechanisms, the consistency of the Brilliant Blue effect across most materials indicates that dye chemistry is a dominant driver of staining outcomes.

The observed material-to-material variability supports the concept that composite composition and microstructure modulate colour stability. Differences in resin matrix chemistry (e.g. relative hydrophilicity of monomer blends), filler loading, particle size distribution, and the quality of filler– matrix coupling can alter water sorption/solubility, surface energy, and micro-porosity, all of which can affect dye uptake and retention [2]. Materials exhibiting smaller shifts after staining and/or better recovery after whitening may be less prone to water-mediated plasticisation and may present fewer sites for chromogen entrapment.

Importantly, the present comparisons are necessarily brand-based because manufacturers use proprietary formulations; nevertheless, the between-material differences can plausibly be interpreted through formulation-level features. Composites with more hydrophilic resin matrices (and higher water sorption) may facilitate dye diffusion and retention, while higher filler loading and improved silane coupling may reduce polymer volume fraction and limit pigment uptake. Optical behaviour can also be influenced by photoinitiator systems, translucency modifiers, and pigment packages, which affect baseline L*/b* and may change how a given dye shifts ∆E00 versus WI_D_. Finally, even when cured against Mylar, subtle differences in surface chemistry and post-cure oxygen inhibition layers can modulate early adsorption of water-soluble dyes.

Notably, Spectra STHV demonstrated comparatively lower susceptibility to Brilliant Blue when judged by the clinically acceptable threshold. This may reflect differences in matrix formulation and/or filler architecture that reduce dye penetration or facilitate removal during rinsing and subsequent treatment. However, because finishing/polishing protocols and surface roughness were not systematically varied in this experiment, the contribution of surface topography to the material effect should be interpreted cautiously.

Mouthwash immersion led to partial reversal of staining (reduced ΔE00 and increased WI_D_), with statistically significant recovery in selected composite–dye combinations. This finding is consistent with *in vitro* evidence that non-hydrogen-peroxide mouthrinses can produce measurable whitening effects, although typically smaller than peroxide-based approaches [16]. It is also in line with studies reporting that commercial mouthrinses may partially recover colour in previously discoloured resin composites [17]. The likely mechanism for the tested formulation is chemical stain control (polyphosphate-based interference with chromogen adhesion plus surfactant-mediated removal) rather than deep oxidative bleaching [9, 18]. The incomplete return to baseline suggests that a portion of the discolouration was not purely superficial. Residual negative shifts in b* in Brilliant-Blue-stained groups at T2 are consistent with persistent blue pigmentation, potentially due to subsurface diffusion or trapping at microscopic defects.

From a clinical perspective, these results suggest whitening mouthrinses may be useful adjuncts for managing early or mild extrinsic discolouration of composite restorations but should not be expected to fully restore baseline shade after intense dye exposure. Where aesthetic demands are high, additional interventions (repolishing, professional cleaning, or restoration replacement) may be required depending on the material and staining source. In addition, clinical outcomes may differ from *in vitro* findings because mouthrinses are commonly used alongside other whitening strategies (e.g. carbamide peroxide), which can enhance overall whitening efficacy [19].

The present findings have practical relevance for material selection and patient counselling. Firstly, because staining was both dye- and material-dependent, clinicians may consider selecting composites with better demonstrated colour stability for highly visible anterior restorations (e.g. diastema closure/recontouring cases), where long-term esthetics are a primary outcome [15]. Secondly, dietary guidance can be tailored: patients with extensive composite restorations or high esthetic expectations should be advised that frequent exposure to strongly coloured drinks/foods – particularly products containing intense blue colorants – may increase the risk of clinically perceptible discolouration, and that discolouration may not be fully reversible with over-the-counter rinses. Practical mitigation strategies include reducing contact time (e.g. limiting sipping duration), rinsing with water after consumption, and maintaining routine professional maintenance. When discolouration occurs, the results support a stepwise management approach: (1) assess whether discolouration is superficial versus intrinsic (e.g. dominant L* reduction vs. persistent chroma/hue shift); (2) initiate conservative measures such as repolishing and stain-control products (polyphosphate- containing rinses/dentifrices) for mild extrinsic staining; and (3) consider replacement in cases with persistent colour mismatch that compromises patient satisfaction. Future clinical trials are needed to determine how well the present immersion model translates to intraoral conditions and to establish evidence-based protocols for the frequency and duration of stain-control mouthrinse use.

Among the tested food dyes, Brilliant Blue produced the most pronounced changes in ΔE₀₀ and WI^D^ values across the majority of materials. This effect was particularly evident in RBF and OBF. RBF is a flowable micro-hybrid composite with a comparatively low filler content (62 wt.%) and a resin matrix based on Bisphenol A-glycidyl methacrylate (Bis-GMA) and Triethylene glycol dimethacrylate (TEGDMA), characteristics that have been associated with greater optical variability. Similarly, OBF, despite being a bulk-fill composite with advanced monomers such as AUDMA and AFM, has a lower filler volume fraction (58.5 vol.%) compared with nanofilled materials. These compositional features may partly explain the larger deviations from baseline colour and whiteness observed in these materials.

Using both ΔE00 and WI_D_ provided complementary information. ΔE00 reflects overall perceptual colour difference, while WI_D_ emphasises changes related to perceived ‘whiteness’, heavily influenced by L* and b*. In this study, dye challenges that shifted b* (particularly toward blue) tended to produce marked WI_D_ changes, and whitening tended to move both metrics toward baseline. However, cases can occur where ΔE00 improves while WI_D_ remains reduced (or vice versa), depending on whether residual changes are dominated by lightness versus chroma/hue components. Reporting both metrics therefore strengthens interpretability for esthetic outcomes in dentistry.

The significant main effects of dye and composite on both ΔE00 and WI_D_ indicate that H01 and H02 should be rejected. The significant effect of experimental stage (including the whitening phase) on both endpoints supports rejection of H03 and H04. Collectively, the data indicate that staining and recovery are dependent on both dye chemistry and composite material, and that the tested whitening mouthwash can mitigate, but not eliminate, food dye-induced discolouration.

This study has limitations typical of *in vitro* discolouration models. Specimens were exposed under static immersion conditions that do not replicate the complexities of the oral environment, including salivary pellicle formation and the buffering/lubricating roles of saliva, dietary cycling, temperature fluctuations, and mechanical abrasion (toothbrushing) [20]. In addition, ageing-related changes in resin composites (e.g. alterations in gloss and colour following artificial ageing) may modify stain susceptibility and stain removal in ways not captured by a short immersion protocol [21]. Surface roughness is also a key determinant of optical behaviour and colour-related performance in contemporary composites; therefore, future work should quantify roughness and relate it to colour outcomes [22]. Finally, clinical exposure to colourants is typically intermittent and occurs in a mixture of beverages/foods rather than single-dye solutions, which may lead to different staining patterns [23]. Although traditional staining agents such as coffee or tea were not included, the use of established perceptibility and clinical acceptability thresholds (ΔE₀₀ = 0.8 and 1.8) enables direct interpretation of staining severity in clinically meaningful terms.

Only one mouthwash and one exposure regimen (7 days) were evaluated; different contact times, concentrations, and formulations (including peroxide-containing rinses and combined protocols) may produce different outcomes [19].

Future research should validate these findings under clinical conditions. In addition, studies linking discolouration and incomplete recovery to measurable material properties (e.g. water sorption/solubility, surface roughness, and ageing-related changes) would help explain the observed material variability and guide evidence-based material selection [21, 24].

## Conclusions

The colour stability and whiteness of dental resin composites are affected by synthetic food dyes in a material- and dye-dependent manner. Whitening mouthwash can partially restore both colour difference and whiteness; however, complete recovery to baseline values is generally not achieved.

## Data Availability

The datasets are available from the corresponding author upon reasonable request.
